# Electrocardiographic Scaling Reveals Differences in Electrocardiogram Interval Durations Between Marine and Terrestrial Mammals

**DOI:** 10.3389/fphys.2021.690029

**Published:** 2021-09-22

**Authors:** Rhea L. Storlund, David A. S. Rosen, Andrew W. Trites

**Affiliations:** ^1^Department of Zoology, University of British Columbia, Vancouver, BC, Canada; ^2^Marine Mammal Research Unit, Institute for the Oceans and Fisheries, University of British Columbia, Vancouver, BC, Canada; ^3^Vancouver Aquarium, Vancouver, BC, Canada

**Keywords:** ECG, marine mammal, heart rate, anesthesia, allometry, cardiac timing, comparative electrophysiology

## Abstract

Although the ability of marine mammals to lower heart rates for extended periods when diving is well documented, it is unclear whether marine mammals have electrophysiological adaptations that extend beyond overall bradycardia. We analyzed electrocardiographic data from 50 species of terrestrial mammals and 19 species of marine mammals to determine whether the electrical activity of the heart differs between these two groups of mammals. We also tested whether physiological state (i.e., anesthetized or conscious) affects electrocardiogram (ECG) parameters. Analyses of ECG waveform morphology (heart rate, P-wave duration, and PQ, PR, QRS, and QT intervals) revealed allometric relationships between body mass and all ECG intervals (as well as heart rate) for both groups of mammals and specific differences in ECG parameters between marine mammals and their terrestrial counterparts. Model outputs indicated that marine mammals had 19% longer P-waves, 24% longer QRS intervals, and 21% shorter QT intervals. In other words, marine mammals had slower atrial and ventricular depolarization, and faster ventricular repolarization than terrestrial mammals. Heart rates and PR intervals were not significantly different between marine and terrestrial mammals, and physiological state did not significantly affect any ECG parameter. On average, ECG interval durations of marine and terrestrial mammals scaled with body mass to the power of 0.21 (range: 0.19–0.23) rather than the expected 0.25—while heart rate scaled with body mass to the power of –0.22 and was greater than the widely accepted –0.25 derived from fractal geometry. Our findings show clear differences between the hearts of terrestrial and marine mammals in terms of cardiac timing that extend beyond diving bradycardia. They also highlight the importance of considering special adaptations (such as breath-hold diving) when analyzing allometric relationships.

## Introduction

Cardiac anatomy and function are widely conserved across mammalian species. Just as heart mass increases with increasing body mass (Prothero, 1979), the timing of cardiac electrical signal conduction is also expected to scale with body mass in mammals because the timing of cardiac filling and contraction must also increase to maintain proper cardiac function (Meijler and Meijler, 2011). Comparative analyses of mammalian electrocardiograms (ECGs) demonstrate that there is a characteristic pattern to electrical depolarization, and that the timing is fairly consistent for all species when body mass is accounted for Günther and Morgado (1997). These patterns not only hold for the allometric relationship between heart rate and body mass, but also hold for other aspects of cardiac electrical activity. This includes the timing of individual components of the PQRST wave that reflect cycles of depolarization and repolarization of various portions of the heart, and produce the characteristic ECG waveform.

Among mammals, the marine species have anatomical and physiological adaptations to breath-hold diving that may cause the electrical activity of their hearts to differ from terrestrial mammals. When diving, the hearts of marine mammals must cope with extreme physiological changes, including bradycardia and vasoconstriction (Blix et al., 1984). Marine mammals regularly achieve lower heart rates while diving than predicted for a similarly sized terrestrial mammal (Fedak et al., 1988; e.g., Castellini and Zenteno-Savin, 1997; Mcdonald and Ponganis, 2014; Goldbogen et al., 2019), as well as higher heart rates than predicted while at the surface (Fedak et al., 1988; Castellini and Zenteno-Savin, 1997; Noren et al., 2012; e.g., Bickett et al., 2019). These heart rate fluctuations make it challenging to predict how ECG intervals and heart rates compare between marine and terrestrial mammals. Differences in the scaling of electrocardiogram (ECG) parameters between marine and terrestrial mammals may be expected due to differences in physiology (e.g., diving bradycardia), gross anatomy (e.g., the general shape of the heart), and tissue composition (e.g., the number and orientation of cardiac muscle fibers, adaptations to the conduction system of the heart).

ECG measurements should be taken under standardized physiological conditions to provide valid comparisons between species (e.g., from calm, healthy individuals). However, in practice, ECGs are recorded under a variety of conditions with one of the most common being under anesthesia. Anesthesia can complicate comparisons because it is known to affect heart rate (Picker et al., 2001; Nishiyama, 2016) and prolong QT intervals (Yildirim et al., 2004). Hence, the effects of anesthesia must be accounted for in any comparison of cardiac electrophysiology.

As with anesthesia, changes in activity state (e.g., rest, exercise, apnea) are also known to affect ECG intervals in terrestrial mammals (e.g., Simoons and Hugenholtz, 1975). However, it is less clear how ECG intervals change with activity in marine mammals, because their apparent resting state at the water’s surface or on land may not be equivalent to that of terrestrial mammals. As such, recording ECGs while marine mammals are submerged may be a more comparative measure (although these are rarely available). This potential difference in activity states of marine and terrestrial mammals may explain the higher-than-expected heart rates recorded from marine mammals at the surface (Fedak et al., 1988; Castellini and Zenteno-Savin, 1997; Noren et al., 2012; e.g., Bickett et al., 2019), and would lead to expected differences in the ECG intervals as well.

Differences in cardiac anatomy provide further reason to investigate ECG scaling in marine and terrestrial mammals. While the hearts of marine and terrestrial mammals are generally anatomically similar (Drabek, 1975; Rowlatt, 1990), the shape of marine mammal hearts may result in different cardiac electrical activity. For example, the broadness of pinniped hearts (Drabek, 1975; Rowlatt, 1990) may increase the duration of ventricular depolarization because the distance the signal must travel is greater, thereby resulting in a longer QRS interval on an ECG. Other changes to the amount and distribution of cardiac muscle will affect how electrical signals travel through the heart, and hence the duration of their ECG intervals.

In addition to gross morphological differences, dissections have also revealed differences in myocardial cell structure that may cause signal conduction in marine mammal hearts to deviate from the typical mammalian pattern. For example, cetaceans such as bowhead whales (*Balaena mysticetus*; Pfeiffer, 1990), sperm whales (*Physeter macrocephalus*; White and Kerr, 1917), pilot whales (*Globicephala* sp.), Atlantic bottlenose dolphins (*Tursiops truncatus*), Pacific white-sided dolphins (*Lagenorhynchus obliquidens*), and Amazon river dolphins (*Inia geoffrensis*; Simpson and Gardner, 1972) have unusually large Purkinje fibers that are thought to increase signal conduction velocity from the atrioventricular (AV) node to the ventricular myocardium (van Nie, 1986). Similarly, Todd fibers found in the right atrial wall of white-beaked dolphins (*Lagenorhynchus albirostris*) are suspected of increasing signal conduction velocity between the sinoatrial (SA) and AV nodes (van Nie, 1987). The function of these specialized conduction tissues is unknown, but it has been suggested that they may be beneficial for rapid heart rate transitions such as those observed in marine mammals as they dive and resurface (van Nie, 1986, 1987), thereby making marine mammals better equipped to rapidly decrease their heart rates when diving and increase their heart rates when surfacing to breathe.

Comparisons of the cardiac electrical activity of marine and terrestrial mammals are needed to identify how differences between these two groups influence scaling arguments. Large scale comparisons across taxa can miss interesting trends because of large interindividual and interspecies variation, especially when all groups are not represented equally, or by neglecting important grouping factors, such as physiological state (i.e., anesthetized or conscious) and ecological group (i.e., marine or terrestrial). Previous allometric analyses of ECG intervals among mammals generally show that scaling is similar between marine and terrestrial mammals (Meijler and van der Tweel, 1986; Meijler, 1990; Noujaim et al., 2004; Meijler and Meijler, 2011). However, the low number of marine mammal species included in these studies prevented direct statistical comparisons between marine and terrestrial mammals. We therefore amassed a dataset of published ECG parameters including 19 species of marine mammals to determine whether cardiac electrophysiology differs between marine and terrestrial mammals when accounting for differences in measurement conditions.

## Materials and Methods

### Meta-Analysis

We amassed an ECG dataset from 83 species (representing over 2,000 individuals) of marine and terrestrial mammals for analysis. The majority of the data were obtained from the scientific literature, but we also added data we collected from three species of marine mammals—Steller sea lions, northern fur seals, and walrus. We extracted values for heart rate, P-wave, and PQ, PR, QRS, and QT interval durations, and recorded information about the source group or individual including age, sex, physiological state (anesthetized or conscious) and body mass, when available. We lumped PQ intervals in with the PR interval comparisons because these intervals are both measured from the onset of atrial depolarization to the onset of ventricular depolarization, despite the slight difference in terminology. We report a single mean or mid-range value for each species that had sample sizes ranging from a single animal to hundreds of individuals (e.g., dogs; Supplementary Table 1). One data point per species per physiological state was used in the analyses to give each species equal weight, independent of sample size.

We categorized the data based on the physiological state of the individual to account for possible effects of anesthesia on cardiac electrophysiology. In our dataset, we classified measurements as being taken under anesthesia when the majority (>90%) of the individuals in the sample were known to be anesthetized. In 22 out of 23 species recorded as anesthetized, all individuals of the species were anesthetized and for the 23rd species, 9 out of 10 individuals were anesthetized. Measurements from 51 species came from individuals that were not anesthetized (i.e., “conscious”). Data from five species [cats (*Felis catus*), dogs (*Canis familiaris*), mice (*Mus musculus*), guinea pigs (*Cavia porcellus*), and northern elephant seals (*Mirounga angustirostris*)] allowed us to report conscious and anesthetized ECG parameters separately. We excluded 24 species from our analyses because no information regarding physiological state (anesthetized or conscious) was available. Our final dataset consists of 69 mammalian species (19 marine and 50 terrestrial), representing 1670 individuals (Supplementary Table 1).

To calculate representative ECG parameters for each species under a specific physiological state, we first calculated means and midranges for each individual source. Averages were preferable, but when these were unavailable, midranges were used as proxies. To combine data from multiple sources, we calculated weighted averages and midranges using sample size as the weighting factor. When sample size was unavailable, we assigned a value of one as the weighting factor because each report had to have come from at least one individual. Therefore, reports from species for which no sample size was stated may be underrepresented in the calculated average for that species.

For terrestrial mammals, we followed the procedure for harvesting ECG data and estimating species’ masses as outlined in Günther and Morgado (1997) with some modifications. Our source for terrestrial ECG data was Grauwiler (1965), who reported ECG parameters for a wide variety of mammals. Often, body mass was not indicated so we estimated body mass by matching reported information about the individual or group, such as age and sex, to corresponding species information from additional literature sources (Supplementary Table 1). When mass estimates could not be informed by age and sex, we used a general average species mass from the available literature. In many cases, estimates of mass were from only one or a few individuals.

For marine mammals, we used ECG data that were previously published for 16 species, to which we added data for three additional species (Steller sea lions, northern fur seals, and a walrus; see Storlund et al., 2021 for detailed methods). Masses for all marine mammal individuals were documented at the time of the ECG recordings either as an estimate (for the large whales) or by direct measurement.

For all species, heart rate data were either explicitly stated or were calculated as 60 divided by the RR interval. Midrange heart rates were calculated and used when averages were not reported. For species that had multiple types of heart rate data available, the reported value used for analysis was selected based on the estimate requiring the fewest number of calculations. Our ranking system from highest to lowest preference was heart rate, heart rate calculated from the RR interval, heart rate midrange, and finally heart rate midrange calculated from RR midrange.

The final data set included ECG parameters published from 1933 to 2021. No data was excluded based on publication date because standards for measuring ECGs have not changed significantly over this time frame (e.g., Lewis, 1913; Wilson et al., 1954; Bickett et al., 2019).

### Statistical Analysis

All statistical analyses were performed using R (v.3.6.3; R Core Team, 2020) and RStudio (v.1.2.5042; RStudio Team, 2020). We fit linear models to test the effects of body mass, ecological group (marine or terrestrial) and physiological state (conscious or anesthetized) on ECG parameters. Body mass and ECG parameters were log_10_-transformed to linearize the data prior to model fit. Initially, we fit saturated three-way interaction models between body mass, ecological group, and physiological state for each ECG parameter. The most parsimonious model for each relationship was determined using visual inspections of interaction plots using the function *plot_model* (package *sjPlot* v.2.8.4; Lüdecke, 2020), followed by likelihood ratio tests using the function *lrtest* (package *lmtest*; Zeileis and Hothorn, 2002). We performed multiple linear regression on the most parsimonious models to evaluate the effect of each independent variable. To check that each model met the assumptions of multiple linear regression analysis, we visually inspected scatterplots of body mass and each ECG interval, Q-Q plots, scatterplots of the predicted values and residuals, and evaluated Variance Inflation Factor values using the function *vif()* (package *car*; Fox and Weisberg, 2019). All data met the assumptions of linearity, multivariate normality, no multicollinearity, and homoscedasticity. Results were assumed to be significant for *p* < 0.05.

## Results

The effects of ecological group, physiological state, body mass, and interactions between these factors were specific to each ECG parameter (Tables 1, 2). All ECG parameters significantly correlated to body mass (Table 1, *p* < 0.001). Heart rate decreased with body mass, while P-wave, PR, QRS, and QT durations increased with body mass (detailed below). Differences between marine and terrestrial mammals were detected in P, QRS, and QT durations, but not in heart rate, or PR durations when mass was accounted for. There were no significant differences between anesthetized and conscious mammals and no interaction effects between body mass, ecological group and physiological state for any ECG parameter.

**TABLE 1:** Multiple linear regression model parameters for relationships between body mass (BM), ecological group (EG), physiological state (PS), and ECG parameters. In these models, the factor EG is 1 for a marine mammal and 0 for a terrestrial mammal and the factor PS is 1 for anesthetized individuals and 0 for conscious individuals.

**Dependent**	***β*_0_**	***l* *o* *g*_10_ *B* *M***	**EG**	**PS**	**BM X PS**	**Adjusted R^2^**	**Model *p-*value**
*log*_10_HR	2.34 ± 0.03^***^	−0.23 ± 0.01^***^	0.03 ± 0.04^NS^	0.03 ± 0.04^NS^	0.05 ± 0.02^NS^	0.86	<0.001
*log*_10_P	−1.53 ± 0.04^***^	0.21 ± 0.02^***^	0.09 ± 0.04^*^	–	–	0.78	<0.001
*log*_10_PR	−1.18 ± 0.02^***^	0.21 ± 0.01^***^	0.00 ± 0.04^NS^	–	–	0.87	<0.001
*log*_10_QRS	−1.53 ± 0.02^***^	0.19 ± 0.01^***^	0.12 ± 0.04^**^	–	–	0.88	<0.001
*log*_10_QT	−0.92 ± 0.2^***^	0.23 ± 0.01^***^	−0.08 ± 0.04^*^	–	–	0.86	<0.001

Values are model coefficients ± SE. Statistical significance of each model coefficient is indicated with *** for p < 0.001, ** for p < 0.01, * for p < 0.05, and NS for p > 0.05.

**TABLE 2:** Linear regressions and allometric equations describing the relationship between body mass (BM) and ECG parameters for mammals separated by ecological group as appropriate.

**Response variable**	**n**	**Ecological group**	**Linear equation of log_10_-transformed data**	**Allometric equation**
HR	73	All mammals	−0.221(BM) +2.346	221.7(BM)*^–^*^0.22^
P	21	Terrestrial mammals	0.209(BM) −1.530	0.029(BM)^0.21^
P	12	Marine mammals	0.208(BM) −1.439	0.036(BM)^0.21^
PR	67	All mammals	0.207(BM) −1.178	0.066(BM)^0.21^
QRS	51	Terrestrial mammals	0.189(BM) −1.530	0.029(BM)^0.19^
QRS	19	Marine mammals	0.189(BM) −1.413	0.039(BM)^0.19^
QT	49	Terrestrial mammals	0.225(BM) −0.918	0.121(BM)^0.23^
QT	17	Marine mammals	0.225(BM) −1.000	0.100(BM)^0.23^

*The number of data points included in the model are also listed (“n”). The units for these equations are kg for BM, bpm for HR, and s for P, PR, QRS, and QT.*

**FIGURE 1:**
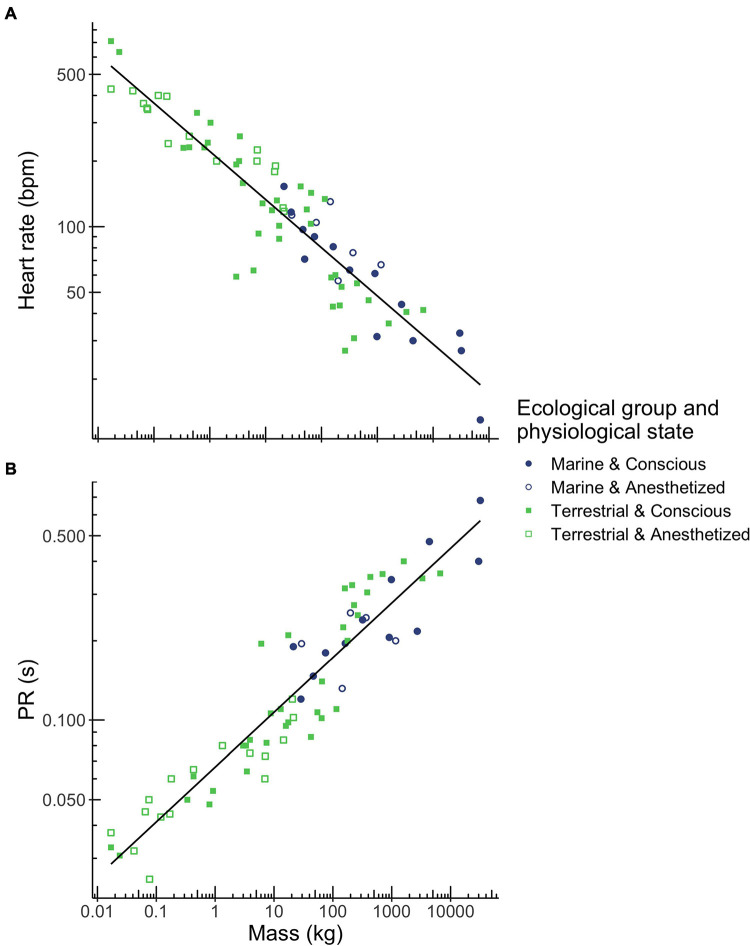
Relationships between body mass and heart rate **(A)**, and PR interval duration **(B)** in mammals. Mass was the best predictor for these three ECG parameters. Ecological group (terrestrial mammals shown in green squares, marine mammals shown in blue circles) and physiological state (conscious mammals indicated by closed squares and circles, anesthetized mammals indicated by open squares and circles) had no effect on heart rate or PR interval. Regression equations for each parameter are provided in Table 2.

Heart rate scaled with body mass to the power of −0.221 and correlated with body mass over a range of 0.017 kg (mouse) to 70,000 kg (blue whale; Figure 1A and Table 2) while PR interval duration scaled with body mass to the power of 0.208 and correlated with body mass over a range of 0.017 kg (mouse) to 32,000 kg (fin whale; Figure 1B and Table 2). Both heart rate and PR interval duration did not vary significantly with ecological group and physiological state (Table 1).

**FIGURE 2:**
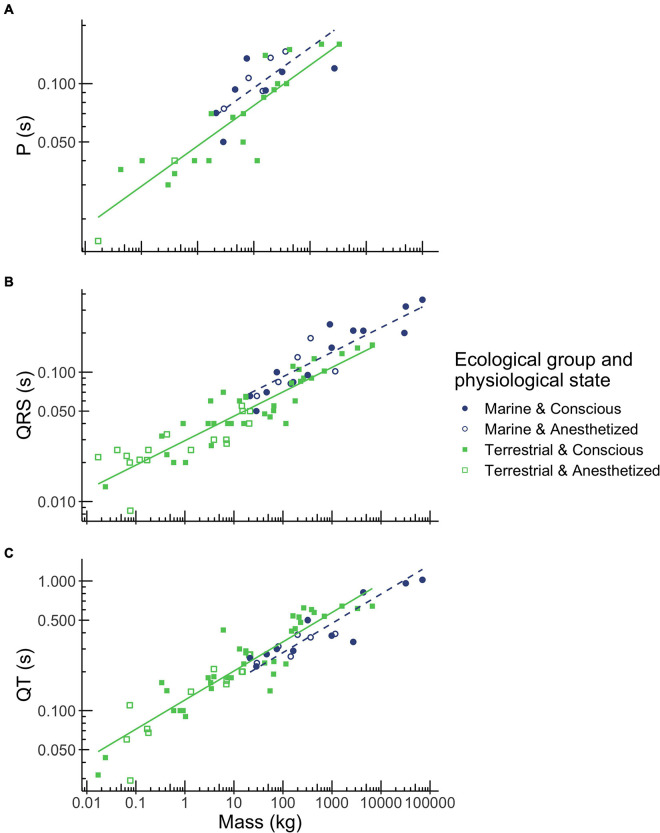
Relationships between body mass, ecological group and P wave duration **(A)**, QRS complex duration **(B)**, and QT interval duration **(C)** in mammals. Mass was the best predictor for these three ECG parameters. However, durations also depended on ecological group (terrestrial mammals shown in green squares, marine mammals shown in blue circles), but not on physiological state (conscious mammals indicated by closed squares and circles, anesthetized mammals indicated by open squares and circles). Regression equations for each parameter are provided in Table 2.

In all mammals, P-wave duration scaled with body mass to the power of 0.209 (Table 2 and Figure 2A). P-wave duration increased with body mass over a range of 0.017 kg (mouse) to 3,320 kg (Asian elephant) and depended on ecological group (Table 1, *p* < 0.001). Marine mammals had 19% longer P-waves than terrestrial mammals when mass was accounted for Table 1, *p* < 0.05. For example, a marine mammal weighing 100 kg would have a P-wave duration of 0.095 s, whereas a terrestrial mammal of the same mass would have a P-wave duration of 0.077 s. P-wave duration did not vary significantly with physiological state (Table 1).

In all mammals, QRS duration scaled with body mass to the power of 0.189 (Table 2 and Figure 2B). QRS duration increased with body mass over a range of 0.017 kg (mouse) to 70,000 kg (blue whale) and depended on ecological group (Table 1, *p* < 0.001). Marine mammals had 24% longer QRS intervals than terrestrial mammals when mass is accounted for. For example, a 100 kg marine mammal would have a QRS complex of 0.092 s, while a terrestrial mammal of the same mass would have a QRS complex of 0.070 s. QRS duration did not vary significantly with physiological state (Table 1).

In all mammals, QT interval duration scaled with body mass to the power of 0.225 (Table 2 and Figure 2C). QT interval duration increased with body mass over a range of 0.17 kg (mouse) to 70,000 kg (blue whale; Table 2, *p* < 0.001) and depended on ecological group (Figure 2C). Marine mammals had 21% shorter QT interval durations than terrestrial mammals when accounting for body mass. For example, a 100 kg marine mammal would have a QT interval of 0.281 s, while a terrestrial mammal of the same mass would have a QT interval of 0.340 s. QT duration did not vary significantly with physiological state (Table 1).

## Discussion

### Marine and Terrestrial Mammal Electrocardiogram Comparison

Our results show that the durations of some components of the ECG waveform differ between marine and terrestrial mammals. Marine mammals have longer P-waves, wider QRS complexes, and shorter QT intervals than terrestrial mammals. These differences indicate that marine mammals have prolonged atrial and ventricular depolarization, and shortened ventricular repolarization compared to terrestrial mammals. In other words, conduction through the atria and ventricles in marine mammals is slower, and repolarization through the ventricles is faster than in terrestrial mammals. These findings likely reflect differences in cardiac anatomy and physiology between these two ecological groups.

We detected several differences between the ECGs of marine and terrestrial mammals despite the large between-species variation observed for all parameters. Only about one quarter of the species we studied fell on or were close to the respective (marine or terrestrial) allometric regression lines, indicating that body mass and ecological group are not the only factors influencing electrical signal conduction through the myocardium. Data points that fall far from the regression lines highlight species-specific differences in cardiac electrophysiology that may relate to unique cardiac adaptations. It is also important to note that the significant differences between marine and terrestrial mammals may not apply to all of the species we grouped within these categories because the hearts of some marine mammals perform more similarly to the hearts of terrestrial mammals and vice versa. Despite this, our comparison showed clear differences in cardiac electrophysiology between marine and terrestrial mammals.

Heart rate comparisons are difficult because they depend on the activity states of the animals being compared. In our study, we found no difference between the heart rates of similar sized marine and terrestrial mammals. This may reflect the challenge of determining what constitutes a “resting heart rate” for marine mammals. For marine mammals, the “resting” cardiac state may be determined by the proportion of time spent diving, surfacing, and (in the case of pinnipeds) on land. Therefore, it may also be important to distinguish between whales, phocids (true seals), and otariids (fur seals and sea lions). For example, whales and seals spend more time diving, so a bradycardic heart rate might be considered their resting state. In contrast, sea lions spend more time hauled-out, so it might be more appropriate to measure their resting heart rate at the surface. It is important to consider this point when interpreting the results of this study as it is possible that some marine mammals were not “at rest” when ECGs were recorded and therefore the data are not directly comparable. Moving forward, defining “rest” in marine mammals will be necessary to improve comparisons of cardiac electrophysiology between ecological groups.

Two of the differences we found in cardiac parameters in our comparison between marine and terrestrial mammals—a longer P-wave and a wider QRS complex—suggest that cardiac anatomy may differ between these two ecological groups. The duration of ECG parameters is influenced by the mass of the myocardium that the electrical signal must pass through because conduction time increases with distance (Lewis, 1920). For example, long QRS intervals are commonly observed in association with left ventricular hypertrophy, especially in elite athletes (Dorn, 2007; Zelenkova and Chomahidze, 2016). Hence, the longer P-waves and QRS complexes of marine mammals may indicate that marine mammals have greater atrial and ventricular myocardial mass or differently shaped cardiac chambers than terrestrial mammals. This is supported by anatomical reports of the broad, dorsoventrally compressed hearts of phocids (Drabek, 1975; Rowlatt, 1990), a shape that would potentially increase the signal conduction distance slowing atrial and ventricular depolarization.

Atrial and ventricular depolarization were slower in marine mammals, but this had no effect on overall cardiac timing (heart rates scaled the same for all mammals) or PR interval duration. A probable explanation is that the enlarged Purkinje fibers and Todd fibers found in some species of cetaceans increase signal conduction velocity through the myocardium (van Nie, 1987, 1988; Pfeiffer, 1990), thereby making up for any possible delay in overall timing. Theoretically, enlarged Todd fibers, such as those found in white-beaked dolphins (van Nie, 1987), could also decrease the timing of the electrical activity to support the longer P-wave and QRS complex despite heart rate remaining the same. However, it is more difficult to predict the effect of Todd fibers on the ECG because conduction from the SA node to the AV node happens concurrently with atrial depolarization.

The third difference we observed—a shorter QT interval—could be indicative of differing activity states of marine and terrestrial mammals when ECGs were recorded. QT intervals are largely determined by heart rate and shorten when heart rate increases (Lecocq et al., 1989). While there were no differences in heart rate between marine and terrestrial mammals, the shortened QT intervals might be further evidence that the heart rates at the surface (where most ECGs were recorded) of some marine mammals are, in fact, elevated above true resting values. In such cases, “resting” heart rates likely occur subsurface in many marine mammal species.

Inherent differences in the timing of cardiac action potentials between marine and terrestrial mammals can also explain the ECG differences we found. The duration of an action potential is determined by time-dependent and voltage-gated membrane currents. Prolonged atrial and ventricular depolarization can result from decreased sodium current, while short QT intervals can result from increased potassium current or decreased calcium current (e.g., Gima and Rudy, 2002; Antzelevitch et al., 2007; Amin et al., 2010; Gintant et al., 2011). Comparisons of marine and terrestrial cardiac myocytes are needed to test this hypothesis.

### Effects of Anesthesia

Anesthesia is known to affect heart rate and QT interval duration, but did not have a noticeable effect on ECG durations in this study. This is not to say that anesthesia does not affect ECG parameters, only that we did not detect any differences in our study. Anesthesia was only retained in the final model describing heart rate, and it did not have a statistically significant effect. Due to the large variation in heart rates observed for a given mass, the effect of anesthesia may not have been noticeable. In addition, anesthetic protocols can have opposing effects on heart rate with some increasing heart rate (Picker et al., 2001) while others decrease heart rate (Ko and Krimins, 2014; Ozeki and Caulkett, 2014). Since the anesthetic agent in many of these studies was not specified, this could also contribute to the lack of observable effect. Anesthetic agents such as isoflurane, desflurane, and sevoflurane are also known to cause QT intervals to increase (Yildirim et al., 2004), but that was not apparent in the current study. In many of the published studies that we took data from, only limited information about the anesthetic protocol used (e.g., type, plane, and duration of anesthesia) was available, which prevented us from undertaking further analyses.

### Mammalian Electrocardiogram Scaling

The ECG scaling exponents we found closely agree with other previously derived cardiac scaling exponents. Günther and Morgado (1997) found that the RR, PQ, QRS, and QT intervals all scaled with body mass to the power of 0.20, while our scalers ranged from 0.19 to 0.23. Noujaim et al. (2004) found that mammalian PR intervals scale to a power of 0.24, comparatively higher than the 0.21 determined in our analysis. The similarities between our results and those of Günther and Morgado (1997) are likely due to the similarity in methods and data sources (e.g., Grauwiler, 1965). Additionally, our inclusion of more data from marine mammal species may explain the lower value of our PR interval scaling exponent compared to that of Noujaim et al. (2004), because many of the species that we added are large bodied, putting them on the far end of the body mass spectrum where their PR intervals could greatly impact the overall scaling relationship.

There is debate regarding the theoretical foundation for how ECG characteristics should scale with body mass. Recent studies examining the scaling of ECG parameters with body size report exponents more consistent with the theoretical one-quarter scaling law (Meijler and Meijler, 2011) than with the simple one-third law predicted by Euclid—suggesting that fractal geometry is a more likely explanation for how ECG parameters scale with body mass than volumetric scaling. Still, our empirically derived scaling exponents are lower than the theoretically predicted exponent for all of the ECG parameters we explored. Although our observed values of ∼±0.21 are close to the widely accepted 0.25 derived from fractal geometry, small deviations in exponents will have large impacts on the estimated range of cardiac measurements for a mammal of a given size.

It is possible that the discrepancy between our data and the theoretical exponents reflect variability in nature, measurement error, and technique and operator variability. It is also possible that the theoretically derived scaling arguments are based on a supposed “average idealized animal” that are simply approximations meant to aid understanding of fundamental biological principles (West and Brown, 2005). Currently, no theoretical mechanism exists to explain our consistent allometric scaling to the power of 0.20.

### Limitations

Comparing marine and terrestrial mammal ECGs is difficult because there are relatively few published ECGs from marine mammal species. To address this challenge, we included data from anesthetized subjects and accounted for the potential bias associated with anesthesia by including physiological state as an independent variable in our models. However, we could only categorize each subject as either “anesthetized” or “conscious” because detailed anesthetic protocols were rarely included in the published reports. This adds uncertainty to our analysis because different types of anesthetic agents (and protocols) affect the heart differently. Despite the potential differences in anesthetic protocols, we did not find any effect of physiological state on any ECG parameter, which suggests that the variation due to anesthetic protocol had less of an effect than variation due to other sources. The potential effects of anesthesia are a limitation that can be overcome in future studies as technology advances and more ECGs are recorded from calm, conscious marine mammals.

Another limitation of our study is the lack of information regarding ECG recording protocols for each subject. The sources we used rarely included information about perceived stress, activity level, limb leads, electrode placement, and measurement protocols. As a result, we assumed that ECG measurements were comparable despite potential differences in protocols. However, our results do not appear to have been undermined by this assumption given that the trends we observed agree with previously described patterns in mammalian ECGs.

Analyzing group differences in allometric relationships can be challenging when the groups being compared have large differences in body mass. In our case, the average mass of terrestrial mammals was small compared to the average mass of marine mammals, and the masses of the two groups only overlapped over a portion of their range. However, we felt it appropriate to retain all of the species in the final model because we could find no indication that ECG parameters from the smallest and largest species were outliers.

## Conclusion

Overall, our study supports previous findings about mammalian ECG interval and heart rate scaling, while also demonstrating the need to consider ecological groups when making comparisons based on allometric relationships. The timing of electrical conduction through the myocardium is altered slightly in marine mammals, probably to maintain the timing of chamber filling and contraction. Without this unique timing, the heart beats of marine mammals would be slowed, which could negatively affect circulation. Clear differences in the cardiac timing of marine mammals are likely the result of anatomical adaptations to diving, rather than these differences being functional adaptations themselves.

## Data Availability Statement

The original contributions presented in the study are included in the article/Supplementary Material, further inquiries can be directed to the corresponding author/s.

## Author Contributions

RS collected, analyzed the ECG data, and wrote the first draft of the manuscript. All authors contributed to the conception and design of the study and contributed to the revisions.

## Conflict of Interest

The authors declare that the research was conducted in the absence of any commercial or financial relationships that could be construed as a potential conflict of interest.

## Publisher’s Note

All claims expressed in this article are solely those of the authors and do not necessarily represent those of their affiliated organizations, or those of the publisher, the editors and the reviewers. Any product that may be evaluated in this article, or claim that may be made by its manufacturer, is not guaranteed or endorsed by the publisher.
