# Supplementary material for: Electrocardiographic Scaling Reveals Differences in Electrocardiogram Interval Durations Between Marine and Terrestrial Mammals
Source: Front Physiol. 2021 Sep 22;12:690029. doi: 10.3389/fphys.2021.690029 (PMC8493095; doi:10.3389/fphys.2021.690029)
Supplement: Supplementary file 1 [file Table_1.pdf]

Supplementary Material

Table S1. Electrocardiographic measurements of mammals. n is the minimum sample size used to calculate average ECG parameters for each species. PS indicates the physiological state of the individuals at the time of the recording, Y = anesthetized, N = conscious. EG indicates the ecological group of the species, either marine (M) or terrestrial (T). HR = heart rate, P = P-wave duration, PR = PR interval, QRS = QRS complex duration, QT = QT interval. ECG sources are references for the ECG data and mass sources are references for the mass data. All terrestrial mammal ECG references were cited in Grauwiler (1965).

Species	Common name	n	Mass (kg)	PS	EG	HR (bpm)	P (s)	PR (s)	QRS (s)	QT (s)	ECG source	Mass source
Balaenoptera musculus	Blue whale	1	70000	N	M	13.0	NA	NA	0.361	1.021	Goldbogen et al. 2019	Goldbogen et al. 2019
Balaenoptera physalus	Fin whale	1	32000	N	M	27.0	NA	0.680	0.320	0.960	Senft & Kanwisher 1960	Senft & Kanwisher 1960
Callorhinus ursinus	Northern fur seal	5	29.38	Y	M	113.0	0.074	0.195	0.066	0.234	Storlund et al. 2021	Storlund et al. 2021
Delphinapterus leucas	Beluga whale	3	986	N	M	31.3	NA	0.341	0.155	0.380	Kanwisher & Ridgway 1983 cited in Kastelein & Meijler 1989, King et al. 1953, Bickett et al. 2019	Kanwisher & Ridgway 1983 cited in Kastelein & Meijler 1989, King et al. 1953, Bickett et al. 2019
Delphinus delphis	Common dolphin	1	50	N	M	71.0	NA	NA	NA	NA	Kanwisher & Ridgway 1983 cited in Kastelein & Meijler 1989	Kanwisher & Ridgway 1983 cited in Kastelein & Meijler 1989

Eschrichtius robustus	Gray whale	1	4363	N	M	30.0	NA	0.475	0.208	0.817	Ponganis & Kooyman 1999	Ponganis & Kooyman 1999
Eumetopias jubatus	Steller sea lion	8	198.8	Y	M	56.5	0.136	0.255	0.130	0.386	Storlund et al. 2021	Storlund et al. 2021
Globicephala macrorhynchus	Pilot whale	1	907	N	M	61.0	NA	0.206	0.233	NA	Bickett et al. 2019	Bickett et al. 2019
Megaptera novaeangliae	Humpback whale	2	30000	N	M	32.5	NA	0.400	0.200	NA	Meijler et al. 1992	Meijler et al. 1992
Mirounga angustirostris	Northern elephant seal	1	75	N	M	90.0	0.135	0.180	0.100	0.300	Hamlin et al. 1972	Hamlin et al. 1972
Mirounga angustirostris	Northern elephant seal	13	1167	Y	M	66.9	NA	0.200	0.102	0.392	Hamlin et al. 1972, Van Citters et al. 1965	Hamlin et al. 1972, Van Citters et al. 1965
Mirounga leonina	Southern elephant seal	24	144.1	Y	M	130.3	0.092	0.132	0.082	0.263	Falabella et al. 1999	Falabella et al. 1999
Odobenus rosmarus	Pacific walrus	1	366.5	Y	M	76.0	0.147	0.245	0.182	0.369	Storlund et al. 2021	Storlund et al. 2021
Orcinus orca	Killer whale	4	2718	N	M	44.0	0.120	0.217	0.209	0.340	Meijler & van der Tweel 1986, Bickett et al. 2019	Meijler & van der Tweel 1986, Bickett et al. 2019
Otaria flavescens	Southern sea lion	13	80.66	Y	M	104.6	0.107	NA	0.084	0.314	Dassis et al. 2016	Dassis et al. 2016
Phoca vitulina	Harbor seal	59	21.3	N	M	153.0	0.071	0.161	0.065	0.255	Hamlin et al. 1972, Fonfara et al. 2015, Kaczmarek et al. 2018	Hamlin et al. 1972, Cottrell et al. 2002, Kaczmarek et al. 2018

Phocoena phocoena	Harbour porpoise	3	28.7	N	M	116.7	0.050	0.120	0.050	0.220	Kastelein & Meijler 1989	Kastelein & Meijler 1989
Trichechus manatus	Manatee	12	320.1	N	M	63.3	0.115	0.240	0.095	0.500	Siegal-Willott et al. 2006	Quiring & Harlan 1953, Etheridge et al. 1985
Tursiops truncatus	Bottlenose dolphin	41	162.2	N	M	81.0	0.092	0.203	0.084	0.290	Harms et al. 2013, Hamlin et al. 1970, Kanwisher & Ridgway 1983 cited in Kastelein & Meijler 1989	Harms et al. 2013, Hamlin et al. 1970, Kanwisher & Ridgway 1983 cited in Kastelein & Meijler 1989
Zalophus californianus	California sea lion	4	46.5	N	M	97.0	0.093	0.147	0.070	0.273	Hamlin et al. 1972, Kaczmarek et al. 2018	Hamlin et al. 1972, Kaczmarek et al. 2018
Acomys cahirinus	Spiny mouse	5	0.065	Y	T	367.5	NA	0.045	0.023	0.060	Grauwiler 1965	Shkolnik & Borut 1969 cited in Heusner et al. 1991
Bos grunniens	Domestic yak	1	15.9	N	T	132.0	0.040	0.095	0.040	0.230	Siegfried 1956	Zi et al. 2004
Camelus bactrianus	Camel	2	433	N	T	55.0	0.150	0.349	0.127	0.572	Grauwiler 1961	Tsogttuya et al. 2009
Camelus dromedarius	Dromedary	1	265	N	T	27.0	0.100	0.250	0.090	0.620	Braun et al. 1958	Schmidt-Nielson et al. (1967) cited in Heusner et al. 1991
Canis aureaus	Jackal	1	8.8	N	T	128.0	0.040	0.106	0.040	0.180	Siegfried 1956	Gittleman 1985
Canis familiaris	Domestic dog	686	64.4	N	T	103.2	0.050	0.102	0.050	0.192	Corticelli 1949, Horwitz et al. 1953, Lannek 1949, Petersen et al. 1951, Schulze et al. 1957,	Gehr et al. 1981, American Kennel Club

											Soave 1954, Sporri 1954, Grauwiler 1961	
Canis familiaris	Domestic dog	106	21.35	Y	T	117.6	NA	0.102	0.050	0.272	Doherty & Hara 1961, Lombard & Witham 1955, Santos & Kittle 1958, Siems et al. 1955	Gehr et al. 1981
Cavia porcellus	Guinea pig	355	0.429	N	T	231.4	0.036	0.061	0.023	0.142	Sporri 1944, Jasinski & Grauwiler 1960, Jasinski & Grauwiler 1960	Gehr et al. 1981
Cavia porcellus	Guinea pig	10	0.429	Y	T	260.0	NA	0.065	0.033	NA	Lombard 1952	Gehr et al. 1981
Choeropsis liberiensis	Pigmy hippopotamus	2	229	N	T	53.0	0.093	0.273	0.085	0.481	Siegfried 1956; Grauwiler 1961	Vendl et al. 2016
Choloepus didactulus	Two-toed sloth	1	6.07	N	T	63.0	NA	0.195	0.070	0.420	Grauwiler 1961	Adam 1999
Didelphis marsupialis	Common opossum	10	1.329	Y	T	200.0	NA	0.080	0.025	0.140	Wilber 1955	McNab (1978a) cited in Heusner et al. 1991
Didermoceros sumatraensis	Sumatran rhinoceros	1	698.1	N	T	46.0	NA	0.358	0.102	0.535	Grauwiler 1961	Dierenfeld 1999
Echidna aculeata	Spiny anteater	2	2.978	N	T	59.0	NA	NA	NA	NA	Grauwiler 1965	Augee et al. 1970
Elephas maximus	Asian elephant	21	3320	N	T	40.6	0.160	0.345	0.154	0.613	White et al. 1938, Sporri (unpublished)	Benedict (1938) cited in Heusner et al. 1991, Hile et al. 1997

Eliomys quercinus	Garden dormouse	1	0.075	Y	T	350.0	NA	0.050	0.020	0.110	Grauwiler 1965	Grauwiler 1965
Equus asinus	Donkey	2	177.5	N	T	60.0	NA	0.200	0.060	0.430	Luisada et al. 1944	Yousef & Dill (1969) cited in Heusner et al. 1991
Felis catus	Domestic cat	57	3.9	N	T	158.5	0.034	0.084	0.040	0.183	Rothlin & Suter 1947, Schinzel 1933, Sporri 1954, Grauwiler 1961	Taylor et al. 1982
Felis catus	Domestic cat	38	3.9	Y	T	NA	0.040	0.075	0.030	0.210	Blok & Boeles 1957	Taylor et al. 1982
Giraffa camelopardalis	Giraffe	1	383	N	T	30.8	0.100	0.305	0.090	0.602	Grauwiler 1961	Gehr et al. 1981
Gorilla gorilla gorilla	Lowland gorilla	1	13	N	T	119.0	NA	0.110	0.060	0.300	Grauwiler 1961	Harcourt et al. 1981
Hapale bacchus	Common marmoset	1	0.337	N	T	230.0	NA	0.050	0.032	0.165	Grauwiler 1965	Araujo et al. 2000
Herpestes ichneumon	Common mongoose	1	2.98	N	T	193.0	0.030	0.080	0.040	0.180	Grauwiler 1961	Palomares & Delibes 1992
Lama glama	llama	1	115	N	T	134.0	0.040	0.110	0.040	0.230	Siegfried 1956	El-Nouty et al. (1978) cited in Heusner et al. 1991
Loxodonta africana	African elephant	10	6654	N	T	41.5	NA	0.360	0.162	0.640	Siegfried 1956, Grauwiler 1961	Crile & Quiring 1940
Lynx rufus	Bobcat	1	15	Y	T	190.0	NA	NA	0.050	0.200	Grauwiler 1965	Brown & Nicoletto 1991

Macaca irus	Crab-eating macaque	12	7.1	Y	T	225.0	NA	0.073	0.028	0.171	DeWaart & Storm 1935, DeWaart et al. 1936	Tokura et al. 1975
Macaca mulatta	Rhesus monkey	NA	3.46	N	T	259.7	NA	0.064	0.027	0.148	Atta & Vanace 1961 cited in Grauwiler	Crile & Quiring 1940
Macropus bennetti	Bennett's kangaroo	7	54.57	N	T	120.0	NA	0.107	0.045	0.142	Sporri 1956	Tyndale-Biscoe & Renfree 1987 cited in Rose et al. 1997
Marmota marmota	Marmot	1	3.35	N	T	200.0	NA	0.080	0.060	0.165	Grauwiler 1961	Armitage et al. 1976
Martes martes	Pine marten	1	0.92	N	T	243.0	NA	0.054	0.040	0.100	Siegfried 1965	Iversen (1972) cited in Heusner et al. 1991
Meles meles	Badger	4	14.5	Y	T	179.0	NA	0.084	0.055	0.201	Johansson 1957	Iversen (1972) cited in Heusner et al. 1991
Meriones shawii shawii	Gerbil	3	0.18	Y	T	240.5	NA	0.060	0.025	0.068	Grauwiler 1965	Grauwiler 1965
Mesocricetus auratus	Hamster	NA	0.120	Y	T	400.0	NA	0.043	0.021	NA	Lombard 1952, Nakamura & Swank 1960	Tegowska & Gebczynski (1975) cited in Heusner et al. 1991
Microtus arvalis	Common vole	11	0.024	N	T	633.2	NA	0.031	0.013	0.044	Grauwiler 1961	Jansky (1959) cited in Heusner et al. 1991
Mus musculus	Mouse	2	0.017	N	T	710.0	NA	0.033	NA	0.032	Grauwiler 1961	Pearson (1947) cited in Heusner et al. 1991

Mus musculus	Mouse	20	0.017	Y	T	428.0	NA	0.038	0.022	NA	Lombard 1952	Pearson (1947) cited in Heusner et al. 1991
Mustela lutreola	European mink	1	0.59	N	T	333.0	NA	NA	0.020	0.100	Siegfried 1956	Gittleman 1985
Mustela putorius	Polecat	1	1.03	N	T	300.0	0.040	NA	0.020	0.090	Siegfried 1956	Gittleman 1985
Nyctereutes procyonides	Racoon dog	1	7.46	N	T	93.0	NA	0.082	0.040	0.180	Siegfried 1956	Gittleman 1985
Okapia johnstoni	Okapi	4	212.5	N	T	43.5	NA	0.325	0.105	0.528	Sporri unpublished data, Grauwiler 1961	Gijzen 1959 & Grzimek 1958 cited in Bodmer & Rabb 1992
Pan troglodytes	Chimpanzee	1	17.5	N	T	101.0	NA	0.098	0.064	0.280	Grauwiler 1961	Taylor & Rowntree 1973 cited in Taylor et al. 1982
Papio ursinus	Baboon	15	20.5	Y	T	122.0	NA	0.120	0.040	0.270	Kaminer 1958	Kaminer 1958 cited in Grauwiler 1965
Peromyscus	Deer mouse	10	0.042	Y	T	420.0	NA	0.032	0.025	NA	Lombard 1952	Heusner et al. 1991
Phacochoerus aethiopicus	Warthog	1	65.32	N	T	143.0	0.070	0.140	0.055	0.240	Siegfried 1956	Crile & Quiring 1940
Phascolomys latifrons	Hairy-nosed wombat	1	17.5	N	T	88.0	0.070	0.210	0.065	0.290	Grauwiler 1965	Grauwiler 1965
Procyon lotor lotor	Raccoon	1	7	Y	T	200.0	NA	0.060	0.030	0.160	Wilber 1955	Brown & Nicoletto 1991

Rattus norvegicus	Rat	NA	0.17	Y	T	396.6	0.015	0.044	0.021	0.072	Beinfield & Lehr 1956, Heise & Kimbel 1955, Hill et al. 1960, Lombard 1952, Normann et al. 1961, Sambhi & White 1960, Schinzel 1933, Werth 1958	Heusner et al. 1991
Rhinoceros unicornis	Indian rhinoceros	2	1600	N	T	36.0	0.160	0.400	0.139	0.640	Siegfried 1956, Grauwiler 1961	Laurie 1982
Saimiri sciurea	Squirrel monkey	NA	0.8	N	T	231.0	NA	0.048	NA	0.100	Graybiel et al. 1969, Hixson et al. 1960	Heusner et al. 1991
Sus domesticus	Domestic pig	52	42.4	N	T	153.1	0.067	0.086	0.048	0.234	Pantridge 1948, Luisada et al. 1944, Zuckermann 1959, Sporri 1954, Miller et al. 1957, Hausmann 1934, Grauwiler 1961	Crile & Quiring 1940, Sporri 1954 & Grauwiler 1961 cited in Grauwiler 1965
Talpa europaea	European mole	NA	0.077	Y	T	345.0	NA	0.025	0.009	0.029	Grauwiler 1965	von Bonin 1937
Tapirus indicus	Malayan tapir	1	160	N	T	43.0	0.140	0.316	0.111	0.539	Grauwiler 1961	von Bonin 1937
Tapirus terrestris	American lowland tapir	2	150	N	T	58.5	0.085	0.225	0.083	0.412	Siegfried 1956	Bodmer 1990

Table S1 References

Adam, P. J. (1999). Choloepus didactylus. *Mamm. Species* 621, 1–8. doi:10.2307/3504332/2600770.

American Kennel Club Breed weight chart. Available at: https://www.akc.org/expert-advice/nutrition/weight-management/breed-weight-chart/.

Araújo, A., Arruda, M. F., Alencar, A. I., Albuquerque, F., Nascimento, M. C., and Yamamoto, M. E. (2000). Body weight of wild and captive common marmosets (*Callithrix jacchus*). *Int. J. Primatol.* 21, 317–324. doi:10.1023/A:1005433722475.

Armitage, K. B., Downhower, J. F., and Svendsen, G. E. (1976). Seasonal changes in weights of marmots. *Am. Midl. Nat.* 96, 36–51.

Augee, A. M. L., Ealey, E. H. M., and Spencer, H. (1970). Biotelemetric studies of temperature regulation and torpor in the echidna, *Tachyglossus aculeatus*. *J. Mammal.* 51, 561–570.

Bickett, N. J., Tift, M. S., St. Leger, J., and Ponganis, P. J. (2019). Heart rates, heart rate profiles, and electrocardiograms in three killer whales, a beluga, and a pilot whale: an exploratory investigation. *Mar. Mammal Sci.* 35, 1112–1132. doi:10.1111/mms.12578.

Bodmer, R. E. (1990). Fruit patch size and frugivory in the lowland tapir (*Tapirus terrestris*). *J. Zool.* 222, 121–128. doi:10.1111/j.1469-7998.1990.tb04034.x.

Bodmer, R. E., and Rabb, G. B. (1992). *Okapia johnstoni*. *Mamm. Species* 422, 1–8.

Bonin, G. von (1937). Brain-weight and body-weight of mammals. *J. Gen. Psychol.* 16, 379–389.

Brown, J. H., and Nicoletto, P. F. (1991). Spatial scaling of species composition: Body masses of North American land mammals. *Am. Soc. Nat.* 138, 1478–1512.

Cottrell, P. E., Jeffries, S., Beck, B., and Ross, P. S. (2002). Growth and development in free-ranging harbor seal (*Phoca vitulina*) pups from southern British Columbia, Canada. *Mar. Mammal Sci.* 18, 721–733. doi:10.1111/j.1748-7692.2002.tb01069.x.

Crile, G., and Quiring, D. P. (1940). A record of the body weight and certain organ and gland weight of 3734 animals. *Ohio J. Sci.* 40, 219–59.

Dassis, M., Rodríguez, D. H., Rodríguez, E., Ponce De León, A., and Castro, E. (2016). The electrocardiogram of anaesthetized southern sea lion (*Otaria flavescens*) females. *J. Vet. Cardiol.* 18, 71–78. doi:10.1016/j.jvc.2015.09.003.

Dierenfeld, E. S., Wildman, R. E. C., and Romo, S. (2000). Feed intake, diet utilization, and composition of browses consumed by the Sumatran rhino (*Dicerorhinus sumatrensis*) in a North American zoo. *Zoo Biol.* 19, 169–180. doi:10.1002/1098-2361(2000)19:3<169::AID-ZOO1>3.0.CO;2-D.

Etheridge, K., Rathbun, G. B., Powell, J. A., and Kochman, H. I. (1985). Consumption of aquatic

plants by the West Indian manatee. *J. Aquat. Plant Manag.* 23, 21–25.

Falabella, V., Campagna, C., and Lewis, M. (1999). Electrocardiography of southern elephant seal (*Mirounga leonina*) weanlings. *J. Zoo Wildl. Med.* 30, 526–531.

Fonfara, S., Casamian-Sorrosal, D., Sundermeyer, J., and Rosenberger, T. (2015). Variations in heart rate and rhythm of harbor seal pups during rehabilitation. *Mar. Mammal Sci.* 31, 998–1013.

Gehr, P., Mwangi, D. K., Ammann, A., Maloiy, G. M. O., Taylor, C. R., and Weibel, E. R. (1981). Design of the mammalian respiratory system. V. Scaling morphometric pulmonary diffusing capacity to body mass: Wild and domestic animals.

Gittleman, J. L. (1985). Carnivore body size: Ecological and taxonomic correlates. *Oecologia* 67, 540–554. doi:10.1007/BF00790026.

Goldbogen, J. A., Cade, D. E., Calambokidis, J., Czapanskiy, M. F., Fahlbusch, J., Friedlaender, A. S., et al. (2019). Extreme bradycardia and tachycardia in the world's largest animal. *PNAS* 116, 25329–25332.

Grauwiler, J. (1965). *Herz und Kreislauf der Säugetiere. Vergleichend-funktionelle Daten von Jules Grauwiler, mit einem Geleitwort von Heinrich Spörri.* Basel: Birkhäuser Verlag.

Hamlin, R. L., Jackson, R. F., Himes, J. A., Pipers, F. S., and Townsend, A. C. (1970). Electrocardiogram of bottle-nosed dolphin (*Tursiops truncatus*). *Am. J. Vet. Res.* 31, 501–505.

Hamlin, R. L., Ridgway, S. H., and Gilmartin, W. G. (1972). Electrocardiogram of pinnipeds. *Am. J. Vet. Res.* 33, 867–875.

Harcourt, A. H., Fossey, D., and Sabater-Pi, J. (1981). Demography of *Gorilla gorilla*. *J. Zool.* 195, 215–233. doi:10.1111/j.1469-7998.1981.tb03460.x.

Harms, C. A., Jensen, E. D., Townsend, F. I., Hansen, L. J., Schwacke, L. H., and Rowles, T. K. (2013). Electrocardiograms of bottlenose dolphins (*Tursiops truncatus*) out of water: habituated collection versus wild postcapture animals. *J. Zoo Wildl. Med.* 44, 972–981. doi:10.1638/2013-0093.1.

Heusner, A. A. (1991). Size and power in mammals. *J. Exp. Biol.* 160, 25–54.

Hile, M. E., Hintz, H. F., and Erb, H. N. (1997). Predicting body weight from body measurements in Asian elephants (*Elephas maximus*). *J. Zoo Wildl. Med.* 28, 424–427.

Kaczmarek, J., Reichmuth, C., McDonald, B. I., Kristensen, J. H., Larson, J., Johansson, F., et al. (2018). Drivers of the dive response in pinnipeds; apnea, submergence or temperature? *J. Exp. Biol.* 221. doi:10.1242/jeb.176545.

Kastelein, R. A., and Meijler, F. L. (1989). Respiratory arrhythmia in the hearts of Harbour porpoises (*Phocoena phocoena*). *Aquat. Mamm.* 15.2, 57–63.

King, R. L., Jenks, J. L., and White, P. D. (1953). The electrocardiogram of a beluga whale.

Circulation 8, 387–393. doi:10.1161/01.CIR.8.3.387.

Laurie, A. (1982). Behavioural ecology of the Greater one-horned rhinoceros (*Rhinoceros unicornis*). *J. Zool.* 196, 307–341. doi:10.1111/j.1469-7998.1982.tb03506.x.

Meijler, F. L., and van der Tweel, L. H. (1986). De elektrocardiogrammen van 10 olifanten en van de orka in Harderwijk. *Ned Tijdschr Geneeskd* 130, 2344–2348.

Meijler, F. L., Wittkampf, F. H. M., Brennen, K. R., Baker, V., Wassenaar, C., and Bakken, E. E. (1992). Electrocardiogram of the humpback whale (*Megaptera novaeangliae*) with specific reference to atrioventricular transmission and ventricular excitation. *J. Am. Coll. Cardiol.* 20, 475–479. doi:10.1016/0735-1097(92)90120-C.

Palomares, F., and Delibes, M. (1992). Some physical and population characteristics of Egyptian mongooses (Herpestes ichneumon L., 1758) in southwestern Spain. *Z. Saugetierkd.* 57, 94–99.

Ponganis, P. J., and Kooyman, G. L. (1999). Heart rate and electrocardiogram characteristics of a young California gray whale (*Eschrichtius robustus*). *Mar. Mammal Sci.* 15, 1198–1207.

Quiring, A. D. P., and Harlan, C. F. (1953). On the anatomy of the manatee. *J. Mammal.* 34, 192–203.

Rose, R. W., Nevison, C. M., and Dixson, A. F. (1997). Testes weight, body weight and mating systems in marsupials and monotremes. *J. Zool.* 243, 523–531. doi:10.1111/j.1469-7998.1997.tb02798.x.

Senft, A. W., and Kanwisher, J. K. (1960). Cardiographic observations on a fin-back whale. *Circ. Res.* 8, 961–964. doi:10.1161/01.RES.8.5.961.

Siegal-Willott, J., Estrada, A., Bonde, R., Wong, A., Estrada, D. J., and Harr, K. (2006). Electrocardiography in two subspecies of manatee (*Trichechus manatus latirostris* and *T. m. manatus*). *J. Zoo Wildl. Med.* 37, 447–453.

Storlund, R. L., Rosen, D. A. S., Margiocco, M., Haulena, M., and Trites, A. W. (2021). Cardiac examinations of anesthetized Steller sea lions (*Eumetopias jubatus*), northern fur seals (*Callorhinus ursinus*), and a walrus (*Odobenus rosmarus*). *J. Zoo Wildl. Med.*

Taylor, C. R., Heglund, N. C., and Maloiy, G. M. D. (1982). Energetics and mechanics of terrestrial locomotion. I. Metabolic energy consumption as a function of speed and size in birds and mammals. *J. Exp. Biol.* 97, 1–21.

Tokura, H., Hara, F., Okada, M., Mekata, F., and Ohsawa, W. (1975). A comparison of thermoregulatory responses in the Japanese macaque (*Macca fuscata*) and the crab-eating macaque (*Macaca irus*) during cold exposure. *Jpn. J. Physiol.* 25, 147–152. Available at: http://www.ncbi.nlm.nih.gov/pubmed/1152303.

Tsogttuya, C., Luvsan, B., and Erdenebileg, U. (2009). *Review of Mongol camel studies.* , eds. B. S. Fisher and A. M. Fisher Dalanzadgad Available at: http://www.adoptamongoliancamel.com.au/wp-content/uploads/2013/12/Review-of-Mongol-camel-studies-.pdf.

Van Citters, R. L., Franklin, D. L., Smith, O. A., Watson, N. W., and Elsner, R. W. (1965). Cardiovascular adaptations to diving in the northern elephant seal *Mirounga angustirostris*. *Comp. Biochem. Physiol.* 16, 267–276. doi:10.1016/0010-406X(65)90324-5.

Vendl, C., Frei, S., Dittmann, M. T., Furrer, S., Ortmann, S., Lawrenz, A., et al. (2016). Methane production by two non-ruminant foregut-fermenting herbivores: The collared peccary (*Pecari tajacu*) and the pygmy hippopotamus (*Hexaprotodon liberiensis*). *Comp. Biochem. Physiol. - Part A Mol. Integr. Physiol.* 191, 107–114. doi:10.1016/j.cbpa.2015.09.021.

Zi, X. D., Zhong, G. H., Wen, Y. L., Zhong, J. C., Liu, C. L., Ni, Y. A., et al. (2004). Growth performance, carcass composition and meat quality of Jiulong-yak (*Bos grunniens*). *Asian-Australian J. Anim. Sci.* 17, 410–414.
